# Revisiting Mcconnell′s Sign: Right Ventricular Infarction in the Absence of Pulmonary Embolism

**DOI:** 10.1155/crcc/2684465

**Published:** 2026-11-09

**Authors:** Yuki Munekata, Tomoyo Saito, Suguru Ishizaka, Takumi Tsuchida, Shuhei Takauji, Yui Shimono, Takao Konishi, Toshiyuki Nagai, Toshihisa Anzai, Takeshi Wada

**Affiliations:** ^1^ Emergency and Critical Care Center Hokkaido University Hospital Sapporo Japan hokudai.ac.jp; ^2^ Department of Emergency Medicine Sunagawa City Medical Center Sunagawa Japan; ^3^ Department of Cardiovascular Medicine Faculty of Medicine and Graduate School of Medicine Hokkaido University Sapporo Japan hokudai.ac.jp; ^4^ Department of Cardiovascular Medicine National Defense Medical College Tokorozawa Japan ndmc.ac.jp

## Abstract

McConnell′s sign, defined as hypokinesis of the right ventricular (RV) midfree wall with preserved apical contractions, was initially considered to be highly specific for acute pulmonary embolism. However, evidence indicates that it may also appear in other conditions involving RV dysfunction. Here, we report a case of McConnell′s sign associated with RV myocardial infarction in the absence of pulmonary embolism. A 74‐year‐old female was successfully resuscitated following cardiac arrest. Echocardiography revealed a dilated RV with moderate tricuspid regurgitation and a regional wall motion pattern characteristic of McConnell′s sign. Contrast‐enhanced computed tomography excluded a pulmonary embolism. Coronary angiography revealed an occluded right coronary artery, which was identified as the culprit lesion, and a chronically occluded left anterior descending artery with collateral flow. Conservative management led to gradual clinical and echocardiographic improvement. This case underscores that McConnell′s sign is not pathognomonic for pulmonary embolism and should be interpreted cautiously.

## 1. Introduction

In 1996, McConnell et al. [1] described a distinct echocardiographic pattern of right ventricular (RV) dysfunction with akinesis of the midfree wall and normal apical motion that is highly specific for acute pulmonary embolism (PE) [2]. However, subsequent case reports have documented McConnell′s sign in conditions other than PE. Herein, we present a case of McConnell′s sign in the absence of PE and review the relevant literature.

## 2. Case Presentation

A 74‐year‐old female experienced cardiac arrest after complaining of back pain. After approximately 2 min of chest compressions and a single defibrillation, circulation spontaneously returned, and the patient was transferred to our emergency department. Her medical history included right‐sided breast cancer, Type B aortic dissection, hypertension, and hyperuricemia. Her vital signs upon arrival included a Glasgow Coma Scale of E1V1M2, blood pressure of 125/85 mmHg, heart rate of 153 bpm, body temperature of 36.3°C, oxygen saturation of 93%, and respiratory rate of 38/min. Her pupils were 2.0 mm in diameter with a loss of light reflex. Her jugular vein was distended; however, respiratory sounds were clear bilaterally, without lower extremity oedema. Electrocardiography (ECG) revealed atrial fibrillation and Q waves in Leads III and aVF; however, no significant ST changes or SIQIIITIII patterns were observed.

Laboratory data on admission showed troponin T at 71 ng/L, creatine kinase‐MB at 31 U/L, aspartate aminotransferase at 164 U/L, and D‐dimer at 3.55 *μ*g/mL. Transthoracic echocardiography (TTE) showed no left ventricular (LV) asynergy, and neither the LV nor the left atrium was dilated. In contrast, the RV was significantly dilated in the presence of moderate tricuspid regurgitation (TR). However, her TR peak gradient was within normal limits, suggesting the absence of pulmonary hypertension (PH). In addition, midbasal RV wall motion showed McConnell′s sign (hypokinesis with intact wall motion in the RV apex; Figure 1A–D and Supplementary Material 1). Immediate contrast‐enhanced computed tomography revealed no findings suggestive of PE.

**Figure 1:**
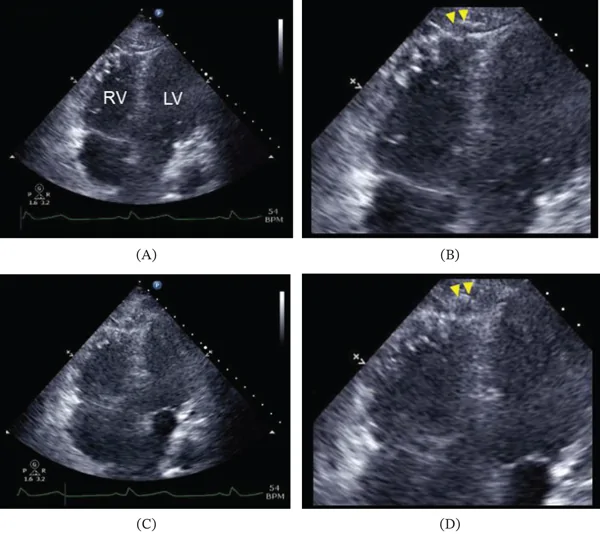
Echocardiographic imaging: (A) the apical four‐chamber view in end‐diastole with (B) a magnified view of the apex, and (C) in end‐systole with (D) corresponding magnified imaging of the apex. Hypokinesis was observed in the mid‐to‐basal segments of the right ventricular free wall during the systolic phase, whereas apical contractility remained preserved, a pattern consistent with McConnell′s sign.

Emergency coronary angiography was subsequently performed, which demonstrated left‐dominant coronary circulation with an occlusion detected in the proximal right coronary artery (RCA), which was estimated to supply a relatively limited myocardial territory (Figure 2A). Additional occlusion was identified in the middle of the left anterior descending (LAD) artery; however, substantial collateral flow originating from the circumflex artery was noted in the delayed phase (Figure 2B). Based on ECG and TTE findings, RCA occlusion was considered the culprit lesion, and percutaneous coronary intervention targeting this area was initiated. However, the procedure was discontinued due to minor procedural complications and angiographic features indicative of a chronically progressive lesion. Although the patient exacerbated right‐sided heart failure after the intervention, her hemodynamic status, including RV wall motion, McConnell′s sign, and TR, gradually improved with conservative medical management. Retrospective quantitative assessment of RV–pulmonary artery (PA) coupling was also performed using the tricuspid annular plane systolic excursion (TAPSE)/systolic pulmonary artery pressure (sPAP) ratio. On hospital Day 1, the TAPSE/sPAP ratio was 0.21 mm/mmHg, indicating marked RV–PA uncoupling. Follow‐up echocardiography at approximately 1 month demonstrated an improvement in the ratio to 0.58 mm/mmHg, accompanied by recovery of RV wall motion and resolution of McConnell′s sign.

**Figure 2:**
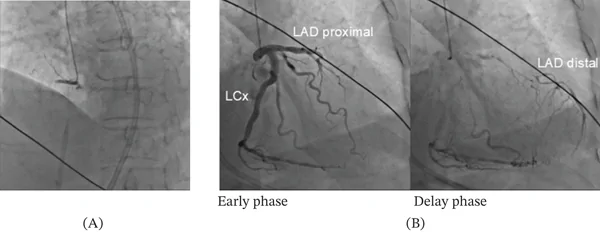
Coronary angiography demonstrated (A) total occlusion of the proximal RCA. (B) Abundant collateral circulation from the left circumflex artery to the distal region of the LAD was confirmed in the delay phase.

## 3. Discussion

We present a case of acute coronary syndrome with McConnell′s sign without PE [3]. The diagnostic utility of McConnell′s sign in PE remains debatable, particularly in terms of sensitivity and specificity. A systematic review and meta‐analysis by Falster et al. [4] reported that McConnell′s sign demonstrated a sensitivity of 29.1% and a specificity of 98.6% for PE diagnosis. Casazza et al. [5] reported that McConnell′s sign was found in 70% of patients with PE and in 67% of those with RV infarction, concluding that it cannot be considered a specific marker of PE in patients with acute RV dysfunction.

To investigate the prevalence of non‐PE cases with McConnell′s sign, we searched PubMed for such case reports by using the keywords “McConnell′s sign.” In addition to the two reported cases of acute RCA infarction [2, 6], McConnell′s sign has been observed in various other conditions, including RCA air embolism [2], cor pulmonale [7], fat embolism syndrome [8], small cell lung cancer [9], amniotic fluid embolism [10], leucostasis associated with chronic myelomonocytic leukemia [11], and pulmonary tumor thrombotic microangiopathy [12]. Similar to our case, these reports further underscore that McConnell′s sign cannot be considered specific for the diagnosis of acute PE.

Three hypotheses have been proposed regarding the pathophysiological basis of echocardiographic findings. The first suggests that tethering of the RV apex to the contracting, often hyperdynamic, LV may account for the preservation of apical motion observed in acute PE [1, 13, 14]. The second hypothesis states that the RV is intrinsically more susceptible to increases in afterload than the LV, causing it to undergo morphological changes in response to the acute increase in afterload caused by PE [15]. In doing so, the RV dilates and adapts toward a spherical shape to more evenly distribute wall stress, with greater outward bulging of the mid‐RV free wall relative to the apex, giving the appearance of free wall hypokinesis [13, 14]. The third hypothesis suggests that localized ischemia of the RV free wall may occur due to increased wall stress in acute PE [1, 13, 14]. Although RV wall motion appears to be relatively preserved, global RV free wall strain and segmental RV free wall strain are lower in all segments, including the apical, mid, and basal regions [16]. Casazza et al. [5] reported that the first and second hypotheses are less likely because the RV apex motion is an inward movement, not just a tethering. They also found that a sudden increase in afterload in RV infarction did not develop in spite of the presence of a normal contracting apex in 67% of their cases. They suggested that the most consistent mechanism to explain McConnell′s sign could be ischemia—functional ischemia of the RV free wall in PE due to a transient increase in wall stress, and necrotic involvement of the RV free wall in RV infarction due to mechanical obstruction of the RCA [1]. Rafie et al. [2] proposed that McConnell′s sign is related to RV ischemia caused by the proximal nature of the RCA occlusion supplying the RV free wall and the wraparound LAD supplying the apex.

Beyond regional wall motion assessment, quantitative evaluation of RV–PA coupling has recently emerged as an important approach to assessing RV performance. RV–PA coupling reflects the relationship between RV contractility and RV afterload and can be estimated noninvasively using the TAPSE/sPAP ratio, calculated from two routinely acquired echocardiographic parameters: TAPSE, obtained by M‐mode imaging of the lateral tricuspid annulus, and sPAP, estimated from the peak TR velocity together with estimated right atrial pressure. Lower TAPSE/sPAP values indicate impaired RV contractile reserve relative to afterload and have been associated with adverse outcomes across a broad spectrum of cardiovascular diseases [17–20]. In the present case, the TAPSE/sPAP ratio improved from 0.21 mm/mmHg during the acute phase to 0.58 mm/mmHg at follow‐up, paralleling the recovery of RV wall motion and resolution of McConnell′s sign. Although the prognostic significance of RV–PA coupling has not been established specifically in RV infarction, our findings suggest that serial assessment of the TAPSE/sPAP ratio may provide complementary quantitative information regarding the severity and recovery of RV dysfunction in patients presenting with McConnell′s sign. Therefore, incorporating RV–PA coupling assessment into routine echocardiographic evaluation may allow a more comprehensive assessment of RV performance and facilitate longitudinal monitoring in everyday clinical practice.

We hypothesized that the underlying mechanisms of McConnell′s sign in non‐PE cases can be broadly classified into either RV infarction or a substantial increase in RV afterload. A possible association between an increase in RV afterload and McConnell′s sign has been reported in non‐RV infarction cases [7–12]. However, RV ischemia may have contributed to these non‐RV infarction cases, as nearly all reported patients demonstrated some degree of elevated serum cardiac enzyme elevation [9, 10, 12]. Only the patient with Cor pulmonale exhibited serum cardiac enzyme levels within the normal range [7], in which McConnell′s sign may have been attributable to morphological changes in the RV associated with an increase in RV afterload. Although the hypothesis that morphological changes secondary to increased afterload contribute to McConnell′s sign cannot be completely excluded, considering the hypothesis proposed by Casazza et al., McConnell′s sign in non‐PE cases may result from RV infarction due to RCA mechanical obstruction or RV functional ischemia with a substantial RV afterload increase.

No clear findings of PH were noted in the present case, and no RV afterload increase was observed. McConnell′s sign in the present case may be attributable to myocardial ischemia resulting from RV infarction in the context of preserved LV function maintained by well‐developed collateral circulation. Additionally, a relative increase in RV afterload secondary to right heart failure may have further contributed to the observed regional wall motion abnormalities.

Finally, the diagnosis of RV infarction may appear less straightforward because neither ST‐segment elevation nor marked cardiac biomarker elevation was observed on admission. However, isolated RV infarction is known to present without classical ECG findings, particularly in patients with left‐dominant coronary circulation [21]. Furthermore, serum troponin concentrations obtained shortly after symptom onset or return of spontaneous circulation may not fully capture the extent of myocardial injury. In the present case, the diagnosis was supported by the combination of proximal RCA occlusion on coronary angiography, regional RV free‐wall dysfunction exhibiting McConnell′s sign, preserved LV systolic function despite chronic LAD occlusion with well‐developed collateral circulation, exclusion of PE, and subsequent recovery of RV function. Collectively, these findings support RV ischemic injury as the most plausible explanation for the observed echocardiographic abnormalities. Another possible explanation for the transient RV dysfunction is postresuscitation myocardial stunning. Cardiac arrest and successful resuscitation are known to induce reversible myocardial dysfunction through global ischemia–reperfusion injury, and transient RV dysfunction following return of spontaneous circulation has occasionally been reported. However, postresuscitation myocardial stunning generally produces diffuse ventricular systolic impairment rather than selective regional RV free‐wall dysfunction. In the present case, preserved LV systolic function, angiographic evidence of proximal RCA occlusion, and the regional distribution of the RV wall motion abnormality support RV ischemia as the principal mechanism, although a contributory effect of myocardial stunning cannot be completely excluded.

This case of RV infarction with McConnell′s sign without PE demonstrates that, although McConnell′s sign was originally introduced as a specific marker of acute PE, it may be observed in a wide variety of conditions that potentially affect RV wall motion. The possible pathophysiological mechanisms underlying McConnell′s sign in non‐PE cases may be RV infarction due to mechanical obstruction of the RCA or RV functional ischemia with a substantial increase in RV afterload.

## Author Contributions

All the authors (Y.M., T.S., S.I., T.T., S.T., Y.S., T.K., T.N., T.A., and T.W.) were involved in the management of the case. Y.M. drafted the manuscript, and Y.M. and S.I. prepared the images, the additional files, and the legends. Y.M. conducted the literature search. T.S., S.I., T.N., T.A., and T.W. provided overall supervision for this case report.

## Funding

No funding was received for this manuscript.

## Disclosure

All authors read and approved the final manuscript. The lead author affirms that this manuscript is an honest, accurate, and transparent account of the study being reported; that no important aspects of the study have been omitted; and that any discrepancies from the study as planned have been explained.

## Ethics Statement

The participant signed an informed consent form and consented to the submission and publication of the case report in the journal. This study was conducted in accordance with the Declaration of Helsinki of the World Medical Association.

## Consent

Written informed consent was obtained from the patient and her family. Written informed consent was obtained from the patient for publication of this case report and accompanying images.

## Conflicts of Interest

The authors declare that they have no conflicts of interest.

## Supporting information


**Supporting Information** Additional supporting information can be found online in the Supporting Information section. Supplementary Material 1: Right ventricle was significantly dilated. The mid‐to‐basal segments of the right ventricular free wall demonstrated hypokinesis, whereas apical contraction was preserved.

## Data Availability

The datasets used and/or analyzed during the current study are available from the corresponding author on reasonable request.
